# The Effects of a Multi-Strain Probiotic Formulation Containing Saffron Extract and Fructooligosaccharides on Anxiety, Depressive Symptoms, Inflammatory Markers, and BDNF: A Double-Blind, Randomized, Placebo-Controlled Pilot Study

**DOI:** 10.3390/microorganisms14102170

**Published:** 2026-09-25

**Authors:** Marina Demetriou, Christos Theleritis, Evangelos Alevyzakis, Efstathios Laskos, Nikolaos Siafakas, Maroulla D. Nikolaki, Ioulia K. Tsetis, Vasiliki Efstathiou, Nikolaos Smyrnis, Panagiotis Ferentinos, Konstantinos Triantafyllou, Nikos C. Stefanis, Emmanouil Rizos

**Affiliations:** 1 Second Department of Psychiatry, “Attikon” University General Hospital, National and Kapodistrian University of Athens, 12462 Athens, Greece; marina-d11@hotmail.com (M.D.); ctheleritis@gmail.com (C.T.); ale.vag77@yahoo.gr (E.A.); v_efstathiou@hotmail.com (V.E.); smyrnis@med.uoa.gr (N.S.); pferentinos@med.uoa.gr (P.F.); 2 Department of Biochemistry and Microbiology, Athens Psychiatric Hospital ‘Dromokaition’, 12461 Athens, Greece; eflaskos@yahoo.gr; 3 Clinical Microbiology Laboratory, “Attikon” University General Hospital, National and Kapodistrian University of Athens, 12462 Athens, Greece; nsiaf@med.uoa.gr; 4 Department of Nutrition and Dietetics, “Attikon” University General Hospital, National and Kapodistrian University of Athens, 12462 Athens, Greece; mnikolaki@med.uoa.gr; 5 Uni-Pharma S.A., 14564 Athens, Greece; jtsetis@uni-pharma.gr; 6 Hepatogastroenterology Unit, Second Department of Internal Medicine and Research Institute, “Attikon” University General Hospital, National and Kapodistrian University of Athens, 12462 Athens, Greece; ktriant@med.uoa.gr; 7 First Department of Psychiatry, Eginition Hospital, School of Medicine, National and Kapodistrian University of Athens, 11528 Athens, Greece; nistefan@med.uoa.gr

**Keywords:** psychobiotics, depression, anxiety, Hospital Anxiety and Depression Scale, neurotrophins, *Lacticaseibacillus*, *Bifidobacterium*, synbiotic

## Abstract

Depression and anxiety are highly prevalent mental health conditions, with growing evidence implicating the gut–brain–immune axis in their pathophysiology. Probiotic supplementation has emerged as a potential psychobiotic intervention, although clinical findings remain inconsistent. This double-blind, randomized, placebo-controlled pilot study assessed the feasibility of a three-month trial and generated preliminary effect estimates for a fixed multi-component formulation. Nineteen psychotropic-naïve adults with a Hospital Anxiety and Depression Scale (HADS) score ≥ 8 on the anxiety and/or depression subscale were randomized to the formulation (n = 9) or matched placebo (n = 10) once daily for three months. The formulation contained *Lacticaseibacillus rhamnosus*, *Bifidobacterium animalis* subsp. *lactis*, *Bifidobacterium breve* and *Bifidobacterium longum* (≥1 × 10^9^ CFU total), 28 mg saffron (Crocus sativus) extract, 100 mg fructooligosaccharides, and 75 mg magnesium—the placebo contained identical excipients and none of the active ingredients. HADS-A, HADS-D, IL-6, TNF-α, CRP, and BDNF were measured at baseline and three months. The cohort was anxiety-predominant (baseline median HADS-A 10.0 [IQR 8.0–11.5] placebo, 11.0 [10.0–12.0] intervention), with baseline HADS-D imbalanced between arms (7.5 [6.25–9.5] vs. 3.0 [2.5–8.0]). Within the intervention arm, BDNF increased over three months (median change +0.27 ng/mL [IQR 0.09–0.84]; Z = −2.429, *p* = 0.015), and HADS-A fell by a median of 1.0 point [IQR −2.0 to 0.5] (*p* = 0.121); within the placebo arm, TNF-α fell (*p* = 0.047), as it also did non-significantly in the intervention arm (*p* = 0.086). No between-group difference in change scores reached significance for any outcome, including BDNF (+0.27 vs. +0.07 ng/mL, *p* = 0.142), HADS-A (−1.0 vs. −0.5 points, *p* = 0.407) and TNF-α (−1.24 vs. −1.08 pg/mL, *p* = 0.838); 21 uncorrected tests were performed. Since the randomized between-group comparison—the only comparison that licenses causal inference—was null throughout, these data support no conclusion about efficacy. Because saffron extract at the dose used has independent antidepressant and anxiolytic activity, no effect can be attributed to the probiotic strains. The study’s usable output are feasibility data and variance estimates for the design of an adequately powered, component-controlled trial.

## 1. Introduction and Background

Growing evidence supports a central role of the gut microbiome in the regulation of central nervous system (CNS) function and emotional behavior, highlighting the gut–brain axis as a key pathway linking gastrointestinal physiology with mental health [1,2,3]. The human gut microbiota, dominated by species from the *Firmicutes, Bacteroidetes, Actinobacteria,* and *Proteobacteria* phyla, contributes to intestinal barrier integrity, immune regulation, and host metabolic homeostasis [4,5]. Communication between the gut microbiome and the CNS occurs through interconnected neural, endocrine, and immune pathways, with the vagus nerve playing a critical role in bottom-up signaling [6,7].

Microbiota-derived metabolites, including short-chain fatty acids, secondary bile acids, and tryptophan-related compounds, act as important mediators of gut–brain communication by influencing enteroendocrine signaling, mucosal immune responses, and systemic inflammation [8]. Some microbial metabolites may also enter the circulation and, in certain cases, cross the blood–brain barrier, potentially exerting direct neuromodulatory effects. Consequently, alterations in gut microbial composition and metabolic activity have been increasingly linked to emotional regulation and vulnerability to psychiatric symptoms.

Microbial dysbiosis has been reported in several psychiatric conditions, including depression, anxiety disorders, psychosis, and is often accompanied by low-grade systemic inflammation, oxidative stress, and altered micronutrient status [8,9,10]. Prebiotics and probiotics have therefore attracted growing interest as potential adjunctive strategies for modulating gut-derived inflammatory and neurobiological pathways. Preclinical studies consistently demonstrate anxiolytic and antidepressant-like effects of probiotic administration, mediated through mechanisms involving immune modulation, hypothalamic–pituitary–adrenal axis regulation, and vagal signaling [11,12]. However, clinical trials in humans have produced heterogeneous findings, underscoring the need for further randomized controlled studies. Meta-analyses suggest that probiotic supplementation may reduce depressive and anxiety symptoms, particularly in clinically diagnosed populations and when used alongside standard pharmacological treatments, with *Lacticaseibacillus* and *Bifidobacterium* strains showing the most consistent effects [13,14]. Nevertheless, evidence remains mixed in subclinical samples, and the biological correlates of these effects, particularly systemic inflammatory markers, are not well characterized.

The present study aimed to evaluate whether probiotic supplementation with a multi-strain formulation containing *Lacticaseibacillus rhamnosus*, *Bifidobacterium animalis* subsp. *lactis*, *Bifidobacterium breve*, and *Bifidobacterium longum* influences symptoms of anxiety and depression in adults, and whether these effects are accompanied by changes in circulating pro-inflammatory markers.

Rather than testing a novel hypothesis about probiotic efficacy, which has been examined in numerous trials and meta-analyses, the present pilot study had two aims: (i) to establish the feasibility of recruiting and retaining psychotropic-naïve, medically ill outpatients with clinically relevant anxiety and/or depressive symptoms into a three-month placebo-controlled trial involving repeated venipuncture, and (ii) to generate preliminary effect-size estimates for a multi-component formulation containing four probiotic strains, saffron extract, fructooligosaccharides, and magnesium, across both psychological (HADS-A, HADS-D) and biological (IL-6, TNF-α, CRP, BDNF) endpoints. The intervention was evaluated as a fixed-dose combination product, as marketed. The design does not permit the contribution of the individual components to be separated, and no component-specific inference is intended or possible. No confirmatory inference is intended.

## 2. Materials and Methods

### 2.1. Study Design

This study is a double-blind, randomized placebo-controlled pilot trial, testing the effect of a multi-component formulation containing probiotics (*Lacticaseibacillus rhamnosus*, *Bifidobacterium animalis* subsp. *lactis*, *Bifidobacterium breve*, *Bifidobacterium longum*), along with saffron extract, fructooligosaccharides, and magnesium, on depressive and/or anxiety symptomatology in 19 participants. Symptom severity was measured on the Hospital Anxiety and Depression Scale at baseline (t0) and three months after the intervention (t1) [8,15]. The HADS uses a four-point Likert scale (0 = not at all, 3 = most of the time), with separate scores for anxiety and depression subscales (0–21 each). Total scores range from 0 to 21 in each subscale, with higher scores indicating greater symptom severity. A score of 0–7 in each subscale is considered normal, a score of 8–10 mild, and a score of 11–21 moderate to severe.

In addition, this pilot study evaluated the effects of this multi-component formulation on inflammatory markers, including interleukin-6 (IL-6), tumor necrosis factor-alpha (TNF-α), and C-reactive protein (CRP), measured from blood samples collected at the same two time points.

Participant recruitment was conducted with the primary psychological outcomes (HADS-A and HADS-D), biomarker outcomes (IL-6, TNF-α, CRP, BDNF), assessment time points, and inclusion/exclusion criteria defined a priori and documented in standardized case report forms prior to this. All participants were provided with a detailed explanation of the study protocol, prior to randomization. After written informed consent was obtained, participants were randomly assigned to two groups. Prospective trial registration was not performed due to the exploratory and feasibility-oriented nature of this pilot randomized controlled study. The study was registered at https://www.isrctn.com/ISRCTN14852868 (accessed on 3 August 2026), under the registration number: ISRCTN14852868.

### 2.2. Study Population

The study population consisted of adults aged 18–85 years who attended the Cardiology, Gastroenterology, or Dermatology Outpatient Clinics of the University General Hospital “Attikon” during the month of June 2023. We assessed all 151 patients who attended the above-mentioned outpatient clinics. Those patients who presented with depressive and/or anxiety symptomatology at the time of assessment, defined as a score ≥ 8 on the Hospital Anxiety and Depression Scale–Depression (HADS-D) and/or the Hospital Anxiety and Depression Scale–Anxiety (HADS-A) and gave written informed consent were eligible to enroll in the study. Meeting the threshold on both sub-scales was not required. Participants were excluded if they did not meet the symptom severity threshold (HADS-D < 8 and HADS-A < 8), were younger than 18 or older than 85 years, or had a current or past psychiatric disorder diagnosis, including schizophrenia spectrum and other psychotic disorders, bipolar and related disorders, other mood disorders, eating disorders, or substance-related and addictive disorders, as defined by the DSM-5. Additional exclusion criteria included the presence of major gastrointestinal disorders at enrollment according to ICD-10 codes K50–K52 and K55–K64 [16], pregnancy within two months prior to enrollment, lactation at the time of enrollment, and exposure within the preceding six months to psychotropic medications (ATC codes N03, N05, N06), immunomodulatory or antineoplastic agents (ATC codes L01, L03, L04), or within the preceding three months to antibiotics, antifungal, antimycobacterial, or antiviral agents, immune sera, or immunoglobulins (ATC codes J01, J02, J04, J05, J06). Participants were also excluded if they had a diagnosis of any active infection within one month prior to enrollment, a confirmed SARS-CoV-2 infection within 35 days prior to enrollment, a clinical indication for initiation of antidepressant or anxiolytic pharmacotherapy at the time of enrollment or had initiated psychotherapy within the 12 weeks preceding or at the time of study entry (Figure 1).

Applying these criteria, 18 out of 19 randomized participants met the threshold on HADS-A (7 met it on HADS-D as well), whereas 12 participants had HADS-D scores in the normal range (0–7). The cohort is therefore anxiety-predominant.

### 2.3. The Intervention

At baseline (t0), all participants underwent a structured clinical psychiatric interview to assess eligibility for study inclusion according to the predefined inclusion and exclusion criteria. During this interview, demographic and clinical data were collected, and additional psychopathology was screened. Subsequently, a validated psychometric instrument, the Hospital Anxiety and Depression Scale (HADS), was administered for the qualitative and quantitative assessment of depressive and anxiety symptoms. At the same time point, a blood sample was collected and processed as whole blood and/or centrifuged serum, either immediately or after storage at −20 °C, for the assessment of hematological parameters and inflammatory and neurotrophic biomarkers, including complete blood count, cytokine levels (IL-6, TNF-α), C-reactive protein (CRP), and plasma brain-derived neurotrophic factor (BDNF). Following informed consent, participants were randomly assigned, in accordance with the double-blind study protocol, to either the intervention group or the control group. Participants in the intervention group received a multi-component formulation, consisting of *Lacticaseibacillus rhamnosus*, *Bifidobacterium animalis* subsp. *lactis*, *Bifidobacterium breve* and *Bifidobacterium longum* (≥1 × 10^9^ CFU total), 28 mg saffron extract, 100 mg fructooligosaccharides, and 75 mg magnesium, whereas those in the control group received a placebo formulation with the same excipients but no active ingredients. We have to clarify that the investigational product was selected because it is a marketed, commercially available formulation supplied as a fixed combination, and the pragmatic question this pilot addressed was whether such a product, in the form consumers actually encounter it, warrants evaluation in an adequately powered trial. The bacterial strains it contains—*Lacticaseibacillus rhamnosus*, *Bifidobacterium animalis* subsp. *lactis*, *B. breve*, and *B. longum*—are among those most frequently studied in relation to affective symptoms. Both formulations were administered orally in the form of granules for suspension, once daily. The manufacturer’s stability data indicate a declared viable count of ≥1 × 10^9^ CFU per stick through end of shelf life when the product was stored at a cool and dry environment with temperature not exceeding 25 °C. Participants were instructed to store the product under the recommended conditions. Follow-up assessment was conducted at three months (t1), at which time the HADS questionnaire was re-administered, and blood samples were collected using the same sampling and processing procedures as at baseline, for repeated measurement of the biomarkers (Table 1).

### 2.4. Randomization

Participants were randomly assigned to either the multi-component formulation intervention group or the placebo control group using a computer-generated randomization sequence. Randomization was performed in blocks, ensuring balanced allocation between the two study arms. The allocation sequence was generated by an independent researcher not involved in participant recruitment, assessment, or data analysis. Each investigational product package was labeled with a unique identification number corresponding to the randomization sequence. Randomization was not stratified by baseline symptom severity; consequences of this will be discussed.

Both the multi-component and placebo formulations were identical in appearance, packaging, taste, and consistency. Treatment allocation was concealed from participants, investigators, clinicians, and study personnel throughout the duration of the study. The blinding codes were securely maintained and were not broken until completion of data analysis, unless required for safety reasons.

### 2.5. Statistical Analysis

Given the exploratory and pilot nature of the present study and the limited sample size, no formal a priori power calculation was performed. Instead, the study was designed to provide preliminary estimates of potential effects and to inform the feasibility and design of future adequately powered randomized trials examining probiotic interventions on psychological and inflammatory outcomes. The analyses reported below are estimation-oriented rather than confirmatory, and are accompanied by measures of precision.

IBM SPSS Statistics for Windows (version 25.0; IBM Corp., Armonk, NY, USA) was used for statistical analyses. The distributional properties of continuous variables were evaluated using the Shapiro–Wilk test. As several of the primary outcome variables deviated from normality, non-parametric statistical methods were employed throughout the analyses. Continuous variables are therefore presented as medians with interquartile ranges (IQR), while categorical variables are summarized as frequencies and percentages.

Baseline group differences were examined to assess comparability between the placebo and intervention groups. Sex distribution was compared using Fisher’s exact test, while continuous baseline variables—including psychological measures and biochemical markers—were compared using the Mann–Whitney U test. Comparisons are reported descriptively. At a total sample of 19, the absence of a statistically significant baseline difference is not strong evidence of comparability, and the difference in median HADS-D between arms is addressed in Section 3.1.

The between group comparison of change scores was designated the primary analysis. To evaluate differences in the magnitude of change between groups, change scores (Δ = t1 − t0) were calculated for each outcome variable. Between-group comparisons of these delta scores were conducted using the Mann–Whitney U test, and are reported together with the Hodges–Lehmann median difference, its 95% confidence interval, and the rank-biserial correlation as a measure of effect size.

Within-group longitudinal changes from baseline (t0) to the three-month follow-up (t1) were evaluated using the Wilcoxon signed-rank test for each treatment group separately. These analyses examined changes in anxiety and depressive symptoms, assessed with the Hospital Anxiety and Depression Scale (HADS), as well as changes in inflammatory and neurotrophic biomarkers, including interleukin-6 (IL-6), tumor necrosis factor-α (TNF-α), brain-derived neurotrophic factor (BDNF), and C-reactive protein (CRP). These analyses are descriptive, and not protected by randomization, as they compare each arm with itself. They are reported for completeness and for the variance estimates they provide, and are not interpreted as evidence of an intervention effect.

As a sensitivity analysis, in view of the baseline imbalance in HADS-D, an analysis of covariance (ANCOVA) of the three-month score on treatment group with the corresponding baseline score as covariate was fitted for HADS-A, HADS-D, and BDNF, together with a rank-based ANCOVA on rank-transformed data as a distribution-free equivalent. Neither model altered the conclusion of the primary analysis: adjusted mean differences (intervention − placebo) were −1.83 for HADS-A (95% CI −5.53 to +1.88; *p* = 0.312), −0.54 for HADS-D (95% CI −3.07 to +1.99; *p* = 0.655), and +0.19 ng/mL for BDNF (95% CI −0.63 to +1.01; *p* = 0.627), and the corresponding rank-based estimates were −0.88 (95% CI −5.72 to +3.96; *p* = 0.704), −0.42 (95% CI −2.87 to +2.04; *p* = 0.725), and +1.40 (95% CI −2.47 to +5.27; *p* = 0.454). Every interval includes zero, and adjustment attenuated rather than strengthened the unadjusted BDNF comparison (*p* = 0.142 unadjusted). The homogeneity-of-slopes assumption was met in all three models (interaction *p* = 0.932, 0.595, and 0.338), but normality of the residuals was rejected for all three (Shapiro–Wilk *p* = 0.001, 0.003, and <0.001), so the rank-based models are the more appropriate.

No adjustment for multiple comparisons was applied. Across six outcomes, 21 statistical tests were performed; under the global null hypothesis, approximately one nominally significant result would be expected by chance alone. Individual *p*-values below the conventional threshold are therefore reported for descriptive completeness and should not, in isolation, be read as evidence of an effect. All statistical tests were two-tailed, and the level of statistical significance was set at *p* < 0.05. Given the small sample size and exploratory design, findings are interpreted cautiously and with primary reference to the randomized between-group comparison and the width of its confidence intervals.

## 3. Results

### 3.1. Participants and Baseline Characteristics

A total of 19 participants were included in this pilot study and were randomly allocated to either the placebo group (n = 10) or the intervention group (n = 9). All 19 participants completed the three-month intervention and provided complete psychological and biochemical data at both time points; no participant was lost to follow-up. Characteristics of the two groups are presented in Table 2.

Statistical comparisons of baseline characteristics are reported in Table 2 and are descriptive. No comparison reached the conventional threshold, but at a total sample size of 19, there is weak evidence of comparability. One baseline difference warrants attention on its own terms: median HADS-D was 7.50 [IQR 6.25–9.5] in the placebo group and 3.00 [2.5–8.0] in the probiotic group, a difference of 4.5 points on a 21-point sub-scale despite a *p*-value of 0.122. This imbalance arose from the eligibility criterion, which required a score of ≥8 on the HADS-Anxiety or the HADS-Depression subscale rather than on both. Of the 19 participants, 18 met the threshold on HADS-A, and the cohort is therefore best described as anxiety-predominant with variable depressive symptom load. The consequence is a floor effect on HADS-D in the probiotic group, which entered the study with a median within the normal range of the sub-scale, and any between-group comparison of change in depressive symptoms should be regarded as uninterpretable.

### 3.2. Primary Analysis: Between-Group Comparison of Change Scores

To evaluate the relative effects of the intervention, between-group comparisons were conducted using delta (Δ) scores, calculated as the change in each variable from baseline to the three-month follow-up; these comparisons constitute the primary analysis. These analyses did not reveal statistically significant differences between the multi-component formulation and placebo groups across any of the examined psychological or biochemical outcomes (all *p* > 0.05), and the accompanying effect sizes and confidence intervals are given in Table 3.

For the two outcomes that showed the largest numerical separation, the estimates were as follows. For Δ BDNF, the Hodges–Lehmann median difference between arms was +0.23 ng/mL (95% CI −0.10 to +0.99; rank-biserial r = 0.40; *p* = 0.142), and for Δ HADS-A, it was −1.00 points (95% CI −4.00 to +1.00; rank-biserial r = −0.22; *p* = 0.407). Descriptive comparisons indicated that the multi-component formulation group showed the more pronounced median improvement in both serum BDNF and anxiety scores, but the imprecision of these estimates does not permit any inference about the direction of effect (Figure 2).

The baseline-adjusted sensitivity analyses described in Section 2.5 did not affect this conclusion. Adjusted for baseline score, the difference between arms was +0.19 ng/mL for BDNF (95% CI −0.63 to +1.01; *p* = 0.627) and −1.83 points for HADS-A (95% CI −5.53 to +1.88; *p* = 0.312); the corresponding rank-based estimates were +1.40 (95% CI −2.47 to +5.27; *p* = 0.454) and −0.88 (95% CI −5.72 to +3.96; *p* = 0.704). Adjustment for baseline therefore attenuated rather than strengthened the non-adjusted BDNF comparison.

### 3.3. Secondary Descriptive Analysis: Within-Group Change

Within-group analyses examining changes from baseline (t0) to the three-month follow-up (t1) are summarized in Table 4. These are uncontrolled within-subject changes in groups of nine and ten participants; they are reported for descriptive completeness and for the variance estimates they contribute to future trial design, and they do not constitute evidence of an intervention effect.

In the multi-component formulation group, an increase in BDNF levels was observed following three months of supplementation (Z = −2.429, *p* = 0.015). This should not be read as a neurobiological effect of the intervention: it is an uncontrolled within-subject change in nine participants, it was one of 21 uncorrected tests, and the corresponding randomized comparison against placebo was null both non-adjusted (*p* = 0.142) and after adjustment for baseline (*p* = 0.627 parametric, *p* = 0.454 rank-based). In addition to the change in BDNF, the multi-component formulation group demonstrated a reduction in anxiety symptoms as measured by the HADS-A sub-scale, which did not reach statistical significance (Z = −1.552, *p* = 0.121). No statistically significant changes were observed within the aforementioned group for depressive symptoms (HADS-D) or for IL-6 and CRP (Figure 3). As noted in Section 3.1, most participants in this group began the study with HADS-D scores within the normal range, so little further improvement on that sub-scale could be expected.

TNF-α declined in both arms over the three-month period, reaching nominal significance in the placebo group (Z = −1.988, *p* = 0.047) and not in the multi-component formulation group (Z = −1.719, *p* = 0.086), while the between-group comparison of change scores showed no difference between them (median Δ −1.08 vs. −1.24 pg/mL, *p* = 0.838). Describing this as an unexpected finding in the placebo arm would contrast two within-group significance thresholds with each other rather than reporting the randomized comparison, and would misrepresent what is a parallel decline in both groups. In the context of baseline values toward the upper end of the assay range (medians 7.20 and 7.97 pg/mL), the substantial within-subject variability of circulating TNF-α, and 21 uncorrected tests, this pattern is most plausibly attributable to regression to the mean and to non-specific effects of trial participation. No significant longitudinal changes were observed within the placebo group for anxiety or depressive symptoms, BDNF, IL-6, or CRP, all of which remained relatively stable throughout the study period.

### 3.4. Summary of Findings

The randomized between-group comparison was null for every psychological and biochemical outcome measured, both npn-adjusted and after adjustment for baseline. The direction of median change favored the multi-component formulation group for BDNF and for anxiety, but the confidence intervals around these estimates are wide and the study, with 19 participants, was underpowered to detect small-to-moderate effects in variables known to exhibit substantial inter-individual variability. These patterns are reported as hypothesis-generating and as a source of variance estimates for the design of an adequately powered trial; they are not evidence of benefit.

## 4. Discussion

The present pilot study explored the effects of a three-month intervention with a fixed multi-component formulation on neurotrophic, inflammatory, and psychological parameters in medically-ill oupatients presenting with clinically relevant anxiety and/or depressive symptoms. The randomized between-group comparison was null for every outcome measured, both unadjusted and after adjustment for baseline, and this is the study’s principal finding. Several within-group changes emerged that are worth describing, most prominently an increase in BDNF in the intervention arm; these are uncontrolled changes in nine participants and are discussed below as observations requiring explanation rather than as effects of the intervention. Preclinical and clinical work has demonstrated that specific probiotic strains can modulate neurotrophin expression and influence stress-related pathways through gut–brain axis mechanisms [17], but as set out in the following paragraph, the present design cannot connect that literature to what was observed here.

The formulation tested contained, alongside the four bacterial strains, 28 mg of saffron extract, 100 mg of fructooligosaccharides, and 75 mg of magnesium. Saffron extract has the most substantial independent evidence base of the four components: randomized trials and meta-analyses of Crocus sativus extract at 28–30 mg/day report reductions in depressive and anxiety symptoms of a magnitude comparable to the changes seen here, with proposed serotonergic mechanisms [18,19]. Fructooligosaccharides act as a prebiotic substrate with their own effects on microbiota composition [20], and magnesium supplementation has itself been examined in affective symptoms. Any change observed in this study may therefore be attributable to saffron, to the prebiotic, to magnesium, to the bacterial strains, or to an interaction among them. The strain-level literature discussed below is presented as background on biological plausibility and is explicitly not a basis for attribution in this trial.

The intervention group also demonstrated a reduction in anxiety symptoms, which did not reach statistical significance within the group and showed no difference from placebo in the randomized comparison. Recent clinical evidence nonetheless supports the plausibility of anxiolytic effects from multi-strain supplementation: a randomized controlled trial published in 2021 reported reductions in perceived stress and improvements in mood-related outcomes [21,22]. These results are in accordance with broader meta-analytic evidence. In addition to that, pooled analyses of randomized controlled trials indicate a reduction in depressive symptoms (SMD −0.96; 95% CI −1.31 to −0.61) in clinically diagnosed populations [1], with benefits appearing strain-specific and *Lacticaseibacillus* and *Bifidobacterium* species producing consistent improvements on self-reported measures such as the Beck Depression Inventory (mean difference −2.69 points; 95% CI −4.22 to −1.16) [11,14]. Clinician-rated outcomes such as the Hamilton Depression Rating Scale show smaller, non-significant effects, likely reflecting limited statistical power or reduced sensitivity in adjunctive trials.

Comparable patterns are evident in anxiety [13]. Meta-analytic evidence indicates a moderate reduction in anxiety symptoms (SMD −0.59; 95% CI −0.98 to −0.19), with the largest effects in clinical or medically ill populations [13,23], whereas across heterogeneous samples pooled effects are smaller and often non-significant (Hedges’ g = −0.12). Among individual strains, *Lacticaseibacillus rhamnosus* has shown promise in preclinical models, though translation to large, well-powered clinical trials remains limited: non-vagotomized mice fed the organism demonstrated decreased anxiety-like behavior [24]. When patients with schizophrenia were administered with *Bifidobacterium breve* A-1, elevated IFN-γ, IL-22, IL-1R, and IL-10 and decreased TNF-α were observed, alongside improved HADS scores [25]. More recent randomized controlled trials suggest that probiotics may offer benefit as adjuncts to standard treatment rather than as standalone agents [21,26]. These findings establish the biological plausibility of the pathways under study; they do not corroborate the present results, which were null.

TNF-α declined in both arms over the three-month period, reaching nominal significance in the placebo group and not in the intervention group, while the randomized comparison of change scores showed no difference between them. Presenting this as an unexpected placebo effect would contrast two within-group thresholds with each other rather than reporting the comparison that randomization protects, and would not describe what is a parallel decline in both groups. Given baseline values toward the upper end of the assay range, the substantial within-subject variability of circulating TNF-α, and 21 uncorrected statistical tests, regression to the mean and non-specific effects of trial participation are the most plausible explanations. The stability of IL-6 and CRP across both arms is consistent with the absence of any systemic anti-inflammatory signal over this timeframe.

The absence of statistically significant between-group differences is unsurprising in a study of this scale. With 19 participants the trial was under-powered to detect small-to-moderate effect sizes, particularly for variables known to exhibit substantial inter-individual variability, and the confidence intervals reported in Table 3 are wide enough to encompass benefit, no effect, and harm. It is worth noting that adjustment for baseline attenuated rather than strengthened the BDNF comparison, which argues against interpreting the within-group increase as a masked treatment effect. Pilot data of this kind should be read for the variance estimates and feasibility information they provide.

Taken together, these findings do not support an effect of the tested formulation on anxiety, depressive symptoms, inflammation, or BDNF, and they illustrate the methodological limits of small pilot studies: susceptibility to type II error, vulnerability to multiplicity, and restricted generalizability. They also identify the design features a definitive trial would require. Future research should employ larger randomized designs with component-controlled arms capable of separating the probiotic strains from saffron and the prebiotic, stratify randomization by baseline symptom severity, incorporate strain-level microbiological outcomes, and consider longer follow-up. Clarifying these relationships may ultimately inform the development of microbiota-targeted strategies for neuropsychological well-being.

## 5. Limitations

Several limitations should be acknowledged. The most consequential concerns the intervention itself: the product tested was a fixed multi-component formulation containing, alongside the four bacterial strains, 28 mg of saffron extract, 100 mg of fructooligosaccharides, and 75 mg of magnesium, each with independent activity relevant to the outcomes measured. No observation reported here can therefore be assigned to any single component. A four-arm factorial design comparing the bacterial strains alone, saffron alone, the full combination, and placebo would be required to disentangle these contributions.

Second, the small sample size substantially limited statistical power and increased the likelihood of Type II error, so that potentially meaningful effects may not have been detected and the generalizability of the findings is restricted. It also precluded subgroup analyses, such as stratification by gender, baseline symptom severity, or inflammatory status. Twenty-one tests were performed across six outcomes without correction for multiplicity, so approximately one nominally significant result would be expected by chance alone; individual *p*-values should not be read in isolation. Findings should be interpreted as exploratory and hypothesis-generating.

Third, baseline HADS-D differed between the arms, the intervention group entering at a median within the normal range of the sub-scale. This followed from an eligibility criterion requiring a score of ≥8 on either sub-scale rather than on both, and the cohort proved anxiety-predominant. The resulting floor effect left essentially no capacity to detect change in depressive symptoms, and baseline-adjusted analysis cannot compensate for such imbalance at this sample size. A confirmatory trial should stratify randomization on HADS-A and HADS-D threshold status.

Fourth, no fecal samples were collected, and gut microbiota composition was not assessed at any time point. Consequently, there is no evidence that the administered strains survived gastrointestinal transit, were present in the colon, or altered the resident microbiota, and species-level detection would in any case not have distinguished administered organisms from resident *B. longum* or *B. breve*. Evidence suggests that bifidobacterial effects on host immune signaling increasingly implicate secreted structures rather than the cell counts alone [27], and recently published protocols in this field pre-specify microbiota composition and function alongside symptom and inflammatory outcomes [28]; measured against that standard, the absence of any microbiological outcome is this study’s principal design deficiency. Incorporating microbiome sequencing, metabolomic profiling, and dietary controls would substantially enhance mechanistic interpretation in future trials [3].

Finally, the three-month intervention may have been too short to capture longer-term psychological or neurotrophic effects; reliance on a self-report instrument may not fully capture subtle changes in emotional processing, and clinician-rated or behavioral measures would be a useful complement. Recruitment challenges related to the burden of biological sample collection and the length of the study period contributed to the small final sample, underscoring the importance of balancing methodological rigor with participant burden when biological endpoints are included.

Taken together, these findings do not support an effect of the tested formulation on anxiety, depressive symptoms, inflammation, or BDNF, and they illustrate the methodological limits of small pilot studies: susceptibility to Type II error, vulnerability to multiplicity, and restricted generalizability. They also identify the design features a definitive trial would require. Future research should employ larger randomized designs with component-controlled arms capable of separating the bacterial strains from saffron and the prebiotic, stratify randomization by baseline symptom severity, incorporate strain-level microbiological outcomes, and consider longer follow-up. Clarifying these relationships may ultimately inform the development of microbiota-targeted strategies for neuropsychological well-being.

## 6. Conclusions

In this double-blind, randomized, placebo-controlled pilot study of 19 psychotropic-naïve outpatients with clinically relevant anxiety and/or depressive symptoms, three months of a fixed multi-component formulation containing four probiotic strains, saffron extract, fructooligosaccharides, and magnesium produced no statistically significant difference from placebo in any psychological or biological outcome. The randomized between-group comparison—the only analysis that supports causal inference—was null for HADS-A, HADS-D, IL-6, TNF-α, CRP, and BDNF alike. A within-arm increase in BDNF and a within-arm decrease in TNF-α reached nominal significance in the intervention and placebo arms respectively, but neither survives the between-group comparison, neither is protected by randomization, and both arose among 21 uncorrected tests; they are reported as descriptive observations and should not be cited as evidence of effect.

What the study does establish is feasibility, and this is its intended contribution. Recruitment of psychotropic-naïve, antibiotic-free, medically ill outpatients meeting a HADS threshold was achievable from a general outpatient population, though at low yield (19 randomized from 151 screened). The observed variability in Δ BDNF and Δ HADS-A provides the variance estimates needed to size a definitive trial. On the strength of these data, we recommend that such a trial be a four-arm component-controlled design (probiotic strains alone, saffron alone, full combination, placebo), stratify randomization by HADS-A and HADS-D threshold status, pre-specify baseline-adjusted ANCOVA of between-group change as the primary analysis, and include strain-specific fecal quantification and microbiota profiling as mandatory secondary outcomes. Until such a trial is conducted, the effect of this formulation on anxiety, depressive symptoms, inflammation, and BDNF should be regarded as unknown.

## Figures and Tables

**Figure 1:**
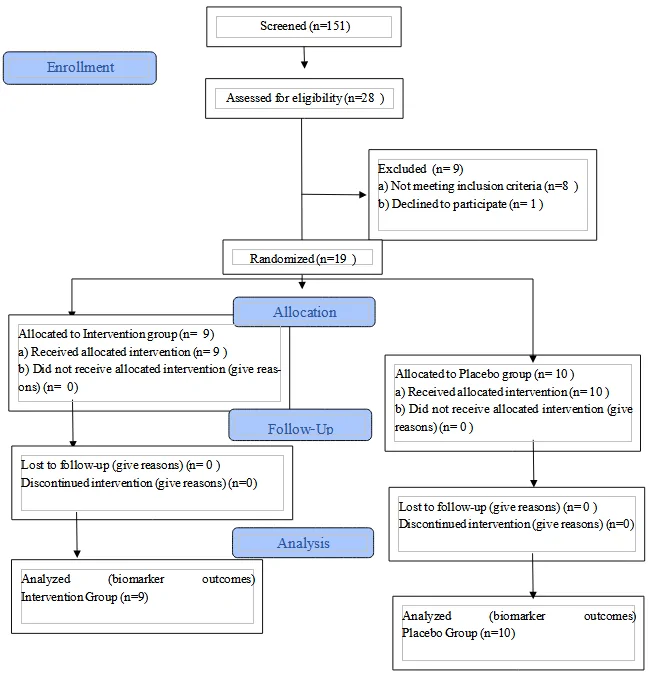
CONSORT flow diagram of participant recruitment, randomization, follow-up, and analysis.

**Figure 2:**
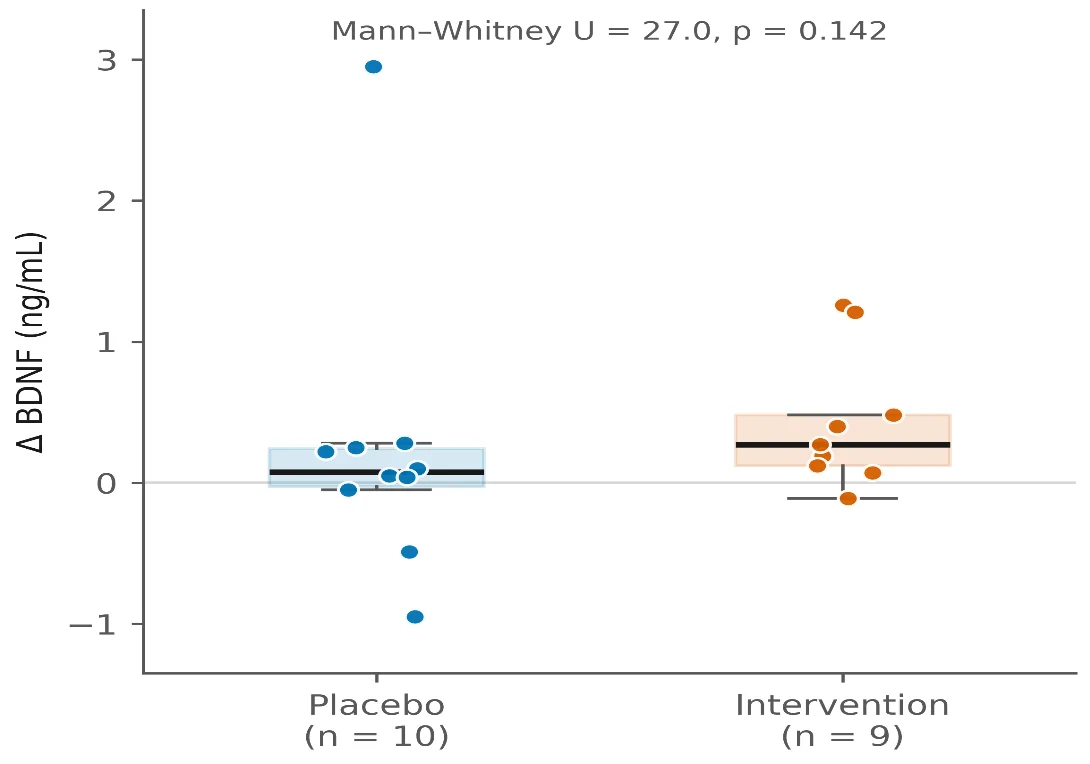
Change in BDNF from baseline to three months (Δ = t1 − t0) in the placebo and intervention arms. Boxes show the median and interquartile range; whiskers extend to 1.5 × IQR; individual participants are overlaid. Between-group comparison by Mann–Whitney U test.

**Figure 3:**
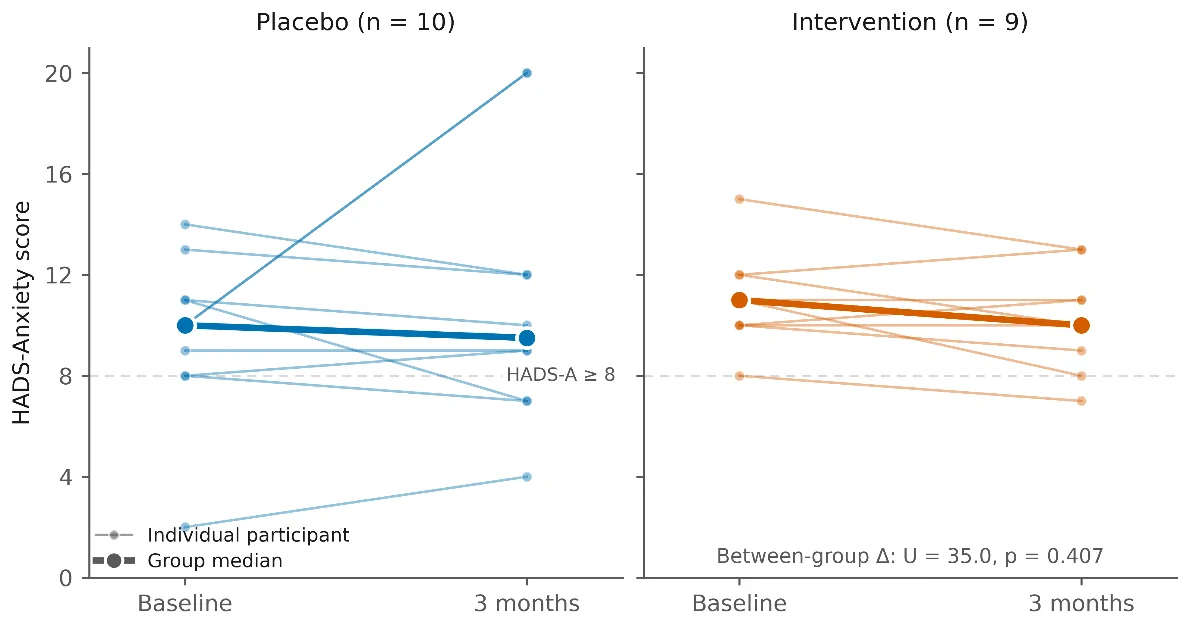
Individual HADS-Anxiety trajectories from baseline to three months. Thin lines show individual participants; the heavy line shows the group median. The dashed line marks the HADS-A inclusion threshold of 8. Between-group comparison of change scores by Mann–Whitney U test.

**Table 1:** The components of the probiotic formulations are presented above; the placebo formulations do not contain any of the active components listed, but include identical excipients.

Components	Concentration (Per Stick)
Magnesium	75 mg (20% NRV)
*Lacticaseibacillus rhamnosus*, *Bifidobacterium animalis* subsp. *lactis*, *Bifidobacterium breve*, *Bifidobacterium longum*	≥1 × 10^9^ CFU
Fructooligosaccharides	100 mg
Saffron extract	28 mg

NRV stands for Nutrient Reference Value.

**Table 2:** Baseline Demographic and Clinical Characteristics of the Study Groups.

Variable	Placebo Group (N = 10)	Intervention Group (N = 9)	*p*-Value
Sex (Female), n (%)	7 (70.0%)	8 (88.9%)	0.582 ^a^
Age (years), Median [IQR]	32.00 [25.0–53.25]	29.00 [25.0–59.0]	0.935 ^b^
HADS-Anxiety, Median [IQR]	10.00 [8.0–11.5]	11.00 [10.0–12.0]	0.077 ^b^
HADS-Depression, Median [IQR]	7.50 [6.25–9.5]	3.00 [2.5–8.0]	0.122 ^b^
IL-6 (pg/mL), Median [IQR]	4.12 [2.00–5.37]	3.71 [1.98–6.65]	0.811 ^b^
TNF-α (pg/mL), Median [IQR]	7.20 [3.36–8.59]	7.97 [5.67–10.76]	0.236 ^b^
BDNF (ng/mL), Median [IQR]	0.91 [0.50–1.32]	0.73 [0.51–1.13]	0.655 ^b^
CRP (mg/dL), Median [IQR]	2.35 [0.70–4.42]	1.20 [0.50–2.60]	0.281 ^b^

IQR: Interquartile Range. ^a^ Fisher’s Exact Test; ^b^ Mann-Whitney U Test. All 19 participants met the inclusion threshold of ≥8 on the HADS-Anxiety subscale.

**Table 3:** Primary analysis: between-group comparison of change scores (Δ = t1 − t0).

Variable (Delta Change)	Placebo (n = 10) Median [IQR]	Intervention (n = 9) Median [IQR]	Mann–Whitney U	*p*-Value	Hodges–Lehmann Difference (95% CI)	Rank-Biserial r
Δ HADS-Anxiety	−0.50 [−1.00–1.75]	−1.00 [−2.0–0.5]	35.00	0.407	−1.00 (−4.00,1.00)	−0.22
Δ HADS-Depression	0.50 [−0.00–2.5]	0.00 [−1.0–0.0]	29.50	0.194	−1.00 (−3.00,1.00)	−0.34
Δ IL-6 (pg/mL)	0.35 [−0.40–1.55]	0.50 [−0.20–5.17]	39.00	0.624	0.46 (−2.22, 6.88)	0.13
Δ TNF-α (pg/mL)	−1.08 [−2.74–0.14]	−1.24 [−1.53–0.76]	43.00	0.870	−0.39 (−2.31, 2.01)	−0.04
Δ BDNF (ng/mL)	0.08 [−0.03–0.24]	0.27 [0.12–0.48]	27.00	0.142	0.23 (−0.10, 0.99)	0.40
Δ CRP (mg/dL)	0.00 [−0.60–0.58]	−0.10 [−0.10–0.00]	41.50	0.774	−0.05 (−0.80, 0.50)	−0.08

Note: Δ (Delta) represents the change score (t2 − t0). *p*-values derived from Mann–Whitney U Test. Data were complete for all 19 randomized participants at both time points.

**Table 4:** Within-Group Changes in Psychological Scores and Biochemical Markers from Baseline to Three Months.

Variable	Placebo Group (N = 10)		Intervention Group (N = 9)	
	Z-Value	*p*-Value	Z-Value	*p*-Value
HADS -Anxiety	−0.299	0.765	−1.552	0.121
HADS-Depression	−1.023	0.306	−0.333	0.739
IL-6 (pg/mL)	−0.816	0.415	−1.007	0.314
TNF-α (pg/mL)	−1.988	0.047	−1.719	0.086
BDNF (ng/mL)	−0.816	0.415	−2.429	0.015
CRP (mg/dL)	−0.411	0.681	−1.611	0.107

## Data Availability

The original contributions presented in this study are included in the article. Further inquiries can be directed to the corresponding author.
